# Supplementary material for: Evaluating Prognosis of Gastrointestinal Metastatic Neuroendocrine Tumors: Constructing a Novel Prognostic Nomogram Based on NETPET Score and Metabolic Parameters from PET/CT Imaging
Source: Pharmaceuticals (Basel). 2024 Mar 14;17(3):373. doi: 10.3390/ph17030373 (PMC10975134; doi:10.3390/ph17030373)
Supplement: Supplementary file 1 [file pharmaceuticals-17-00373-s001.zip › Supplementaryt-done.pdf]

Supplementray Table S1. NRI, IDI, and AUC of the grading systems

	1 year OS		2 year OS		3 year OS		6-month PFS		12-month PFS		18-month PFS	
	**Estimate (95% CI)**	**P value**	**Estimate (95% CI)**	**P value**	**Estimate (95% CI)**	**P value**	**Estimate (95% CI)**	**P value**	**Estimate (95% CI)**	**P value**	**Estimate (95% CI)**	**P value**
**AUC**												
**D grade**	0.812 (0.736-0.889)	Reference	0.786 (0.717-0.855)	Reference	0.802 (0.718-0.889)	Reference	0.830 (0.758 - 0.902)	Reference	0.810 (0.754 - 0.865)	Reference	0.802 (0.748 - 0.856)	Reference
**F grade**	0.700 (0.576-0.824)	0.061	0.797 (0.725-0.868)	0.743	0.792 (0.706-0.877)	0.810	0.697 (0.613 - 0.781)	0.001	0.690 (0.623 - 0.757)	<0.001	0.690 (0.625 - 0.756)	0.001
**S grade**	0.609 (0.476-0.742)	0.002	0.552 (0.462-0.642)	<0.001	0.502 (0.400-0.605)	<0.001	0.657 (0.557 - 0.757)	0.002	0.589 (0.522 - 0.656)	<0.001	0.532 (0.464 - 0.600)	<0.001
**WHO grade**	0.726 (0.623-0.829)	0.100	0.653 (0.580-0.727)	0.003	0.647 (0.600-0.739)	0.005	0.665 (0.573 - 0.756)	0.003	0.613 (0.553 - 0.674)	<0.001	0.632 (0.572 - 0.692)	<0.001
**NRI**												
**D grade**	Reference		Reference		Reference		Reference		Reference		Reference	
**F grade**	0.017 (-0.127 - 0.160)	0.820	-0.120 (-0.295 - 0.055)	0.178	-0.042 (-0.164 - 0.080)	0.500	-0.311 (-0.537 - - 0.085)	0.007	-0.311 (-0.537 - - 0.085)	0.007	-0.316 (-0.493 - - 0.140)	<0.001
**S grade**	-0.345 (-0.471 - - 0.218)	<0.001	-0.639 (-0.819 - - 0.459)	<0.001	-0.306 (-0.450 - - 0.162)	<0.001	-0.464 (-0.651 - - 0.276)	<0.001	-0.368 (-0.505 - - 0.232)	<0.001	-0.700 (-0.926 - - 0.470)	<0.001
**WHO grade**	-0.135 (-0.265 - - 0.005)	0.041	-0.525 (-0.711 - - 0.340)	<0.001	-0.194 (-0.335 - - 0.053)	0.007	-0.135 (-0.265 - - 0.005)	0.041	-0.135 (-0.265 - - 0.005)	0.041	-0.497 (-0.715 - - 0.280)	<0.001
**IDI**												
**D grade**	Reference		Reference		Reference		Reference		Reference		Reference	
**F grade**	-0.033 (-0.054 - - 0.013)	0.001	-0.072 (-0.118 - - 0.027)	0.002	-0.086 (-0.145 - - 0.026)	0.005	-0.106 (-0.150 - - 0.061)	<0.001	-0.146 (-0.207 - - 0.085)	<0.001	-0.101 (-0.148 - - 0.053)	<0.001
**S grade**	-0.071 (-0.092 - - 0.045)	<0.001	-0.166 (-0.215 - - 0.118)	<0.001	-0.223 (-0.287 - - 0.160)	<0.001	-0.146 (-0.192 - - 0.010)	<0.001	-0.225 (-0.290 - - 0.160)	<0.001	-0.189 (-0.242 - - 0.136)	<0.001
**WHO grade**	-0.020 (-0.049 - 0.009)	0.172	-0.070 (-0.130 - - 0.011)	0.020	-0.115 (-0.188 - - 0.041)	0.002	-0.084 (-0.134 - - 0.034)	0.001	-0.142 (-0.213 - - 0.071)	<0.001	-0.124 (-0.182 - - 0.066)	<0.001

D: dual 18F-FDG and 68Ga-DOTANOC PET-CT semiquantitive grade; F: single 18F-FDG PET-CT semiquantitive grade; S: single 68Ga-DOTANOC PET-CT semiquantitive grade;

Supplementray Table S2. NRI, IDI, and C-index of the Grading Systems for OS and PFS in Traing Cohort

	1 year OS		2 year OS		3 year OS		6-month PFS		12-month PFS		18-month PFS	
	**Estimate (95% CI)**	**P value**	**Estimate (95% CI)**	**P value**	**Estimate (95% CI)**	**P value**	**Estimate (95% CI)**	**P value**	**Estimate (95% CI)**	**P value**	**Estimate (95% CI)**	**P value**
**AUC**												
**Nomogram**	0.873 (0.797-0.949)	Reference	0.823 (0.733-0.913)	Reference	0.892 (0.806-0.950)	Reference	0.853 (0.755-0.952)	Reference	0.803 (0.729-0.877)	Reference	0.840 (0.772-0.908)	Reference
**D grade**	0.810 (0.721-0.898)	0.034	0.770 (0.677-0.862)	0.032	0.837 (0.747-0.927)	0.018	0.810 (0.705-0.916)	0.062	0.760 (0.684-0.836)	0.044	0.777 (0.705-0.848)	0.012
**F grade**	0.711 (0.575-0.846)	<0.001	0.770 (0.673-0.867)	0.121	0.800 (0.700-0.903)	0.077	0.651 (0.537-0.764)	<0.001	0.658 (0.573-0.734)	<0.001	0.671 (0.586-0.756)	<0.001
**WHO grade**	0.731 (0.613-0.849)	<0.001	0.653 (0.567-0.739)	<0.001	0.681 (0.575-0.787)	<0.001	0.700 (0.594-0.807)	0.009	0.656 (0.588-0.724)	<0.001	0.653 (0.583-0.723)	<0.001
**NRI**												
**Nomogram**	Reference		Reference		Reference		Reference			Reference		
**D grade**	-0.225 (-0.388 - -0.062)	0.007	-0.074 (-0.203 - 0.055)	0.263	-0.326 (-0.532 - -0.121)	0.002	-0.102 (-0.193 - -0.010)	0.030	-0.169 (-0.371 - 0.034)	0.102	-0.114 (-0.352 - 0.124)	0.347
**F grade**	-0.289 (-0.443 - -0.134)	<0.001	-0.605 (-0.812 - -0.398)	<0.001	-0.370 (-0.521 - -0.219)	<0.001	-0.280 (-0.514 - -0.045)	<0.001	-0.488 (-0.740 - -0.235)	<0.001	-0.608 (-0.799 - -0.417)	<0.001

**WHO grade**	-0.367 (-0.532 - -0.202)	<0.001	-0.430 (-0.647 - -0.213)	<0.001	-0.468 (-0.694 - -0.242)	<0.001	-0.273 (-0.362 - -0.185)	<0.001	-0.294 （ -0.503 - -0.085）	0.006	-0.534 (-0.902 - -0.167)	0.004
**IDI Nomogram**	Reference		Reference		Reference		Reference			Reference		
**D grade**	-0.028 (-0.0549 - -0.001)	0.042	-0.046 (-0.089 - -0.002)	0.039	-0.058 (-0.109 - -0.007)	0.027	-0.048 (-0.076 - -0.021)	<0.001	-0.071 (-0.114 - -0.027)	0.001	-0.074 (-0.130 - -0.018)	0.010
**F grade**	-0.086 (-0.119 - -0.052)	<0.001	-0.154 (-0.210 - -0.099)	<0.001	-0.19 (-0.257 - -0.124)	<0.001	-0.128 (-0.172 - -0.084)	0.019	-0.186 (-0.244 - -0.128)	<0.001	-0.074 (-0.130 - -0.018)	<0.001
**WHO grade**	-0.061 (-0.093 - -0.030)	<0.001	-0.124 (-0.182 - -0.066)	<0.001	-0.172 (-0.249 - -0.094)	<0.001	-0.093 (-0.127 - -0.058)	<0.001	-0.146 (-0.203 - -0.088)	<0.001	-0.139 （ -0.209 - -0.067）	<0.001

D: dual 18F-FDG and 68Ga-DOTANOC PET-CT semiquantitive grade; F: single 18F-FDG PET-CT semiquantitive grade; S: single 68Ga-DOTANOC PET-CT semiquantitive grade;

Supplementtray Table S3. NRI, IDI, and C-index of the Grading Systems for OS and PFS in Internal Validation Cohort

	**1 year OS**		**2 year OS**		**3 year OS**		**6-month PFS**		**12-month PFS**		**18-month PFS**	
	**Estimate (95% CI)**	**P value**	**Estimate (95% CI)**	**P value**	**Estimate (95% CI)**	**P value**	**Estimate (95% CI)**	**P value**	**Estimate (95% CI)**	**P value**	**Estimate (95% CI)**	**P value**
**AUC**												
**Nomogram**	0.889 (0.793-0.986)	Reference	0.892 (0.808-0.975)	Reference	0.821 (0.673-0.969)	Reference	0.899 (0.820-0.978)	Reference	0.919 (0.858-0.980)	Reference	0.914 (0.815-0.977)	Reference
**D grade**	0.826 (0.675-0.977)	0.753	0.815 (0.712-0.918)	0.188	0.745 (0.584-0.906)	0.109	0.860 (0.779-0.942)	0.124	0.899 (0.829-0.968)	0.469	0.852 (0.776-0.929)	0.040
**F grade**	0.640 (0.336-0.944)	0.050	0.832 (0.726-0.937)	0.692	0.779 (0.630-0.927)	0.868	0.759 (0.642-0.875)	0.004	0.732 (0.621-0.842)	<0.001	0.717 (0.617-0.818)	<0.001
**WHO grade**	0.703 (0.487-0.919)	0.236	0.654 (0.518-0.790)	0.003	0.588 (0.427-0.794)	0.028	0.606 (0.443-0.769)	<0.001	0.528 (0.413-0.643)	<0.001	0.593 (0.482-0.704)	<0.001
**NRI**												
**Nomogram**	Reference		Reference		Reference							
**D grade**	-0.164 (-0.382 - 0.054)	0.141	-0.217 (-0.496 - 0.061)	0.126	-0.321 (-0.550 - -0.092)	0.006	0 (-0.110 - 0.110)	1.000	-0.541 (-0.885 - -0.197)	0.002	-0.075 (-0.579 - 0.430)	0.772
**F grade**	-0.008 (-0.256 - 0.239)	0.947	-0.014 (-0.287 - 0.259)	0.919	-0.312 (-0.545 - -0.078)	0.009	-0.285 (-0.652 - 0.081)	0.127	-0.950 (-1.259 - -0.640)	<0.001	-0.675 (-1.014 - -0.337)	<0.001
**WHO grade**	-0.285 (-0.484 - -0.086)	0.005	-0.804 (-1.121 - -0.488)	<0.001	-0.589 (-0.870 - -0.307)	<0.001	-0.224 (-0.340 - -0.108)	<0.001	-0.715 (-1.104 - -0.325)	<0.001	-0.784 (-1.312 - -0.255)	0.004
**IDI**												
**Nomogram**	Reference		Reference		Reference							
**D grade**	-0.063 (-0.119 - -0.006)	0.029	-0.151 (-0.258 - -0.045)	0.005	-0.141 (-0.245 - -0.036)	0.009	-0.041 (-0.083 - 0.001)	0.057	-0.085 (-0.185 - 0.015)	0.097	-0.090 (-0.2493 - -0.069)	<0.001
**F grade**	-0.066 (-0.123 - -0.008)	0.025	-0.160 (-0.266 - -0.054)	0.003	-0.152 (-0.256 - -0.047)	0.004	-0.167 (-0.249 - -0.085)	<0.001	-0.324 (-0.463 - -0.185)	<0.001	-0.303 (-0.474 - -0.132)	<0.001
**WHO grade**	-0.065 (-0.096 - -0.033)	<0.001	-0.216(-0.305 - -0.127)	<0.001	-0.268 (-0.388 - -0.149)	<0.001	-0.148 (-0.211 - -0.086)	<0.001	-0.295 (-0.415 - -0.175)	<0.001	-0.279 (-0.458 - -0.100)	0.004

D: dual 18F-FDG and 68Ga-DOTANOC PET-CT semiquantitive grade; F: single 18F-FDG PET-CT semiquantitive grade; S: single 68Ga-DOTANOC PET-CT semiquantitive grade;

Supplementary Table S4. C-index (95% CI) of Nomogram from Subgroup Analysis by Primary Site

	Train		internal test	
**Original**	**OS**	**PFS**	**OS**	**PFS**
**Stomach**	0.78 (0.60-0.96)	1.00	0.79 (0.70-0.89)	0.84 (0.60-1.00)
**Small intestine**	0.87 (0.78-0.96)	0.82 (0.72-0.92)	0.77 (0.66-0.87)	1.00
**Colorectum**	0.82 (0.73-0.92)	0.85 (0.81-0.90)	0.72 (0.65-0.79)	0.85 (0.75-0.95)

Supplementary Table S5. C-index (95% CI) of Nomogram from Subgroup Analysis by Treatment

	Train		internal test	
**Treatment**	**OS**	**PFS**	**OS**	**PFS**
**Surgery**	0.83 (0.75-0.91)	0.70 (0.63-0.77)	0.88 (0.80-0.97)	0.79 (0.73-0.86)
**Without Surgery**	0.79 (0.70-0.88)	0.76 (0.70-0.69)	0.82 (0.70-0.94)	0.84 (0.77-0.91)

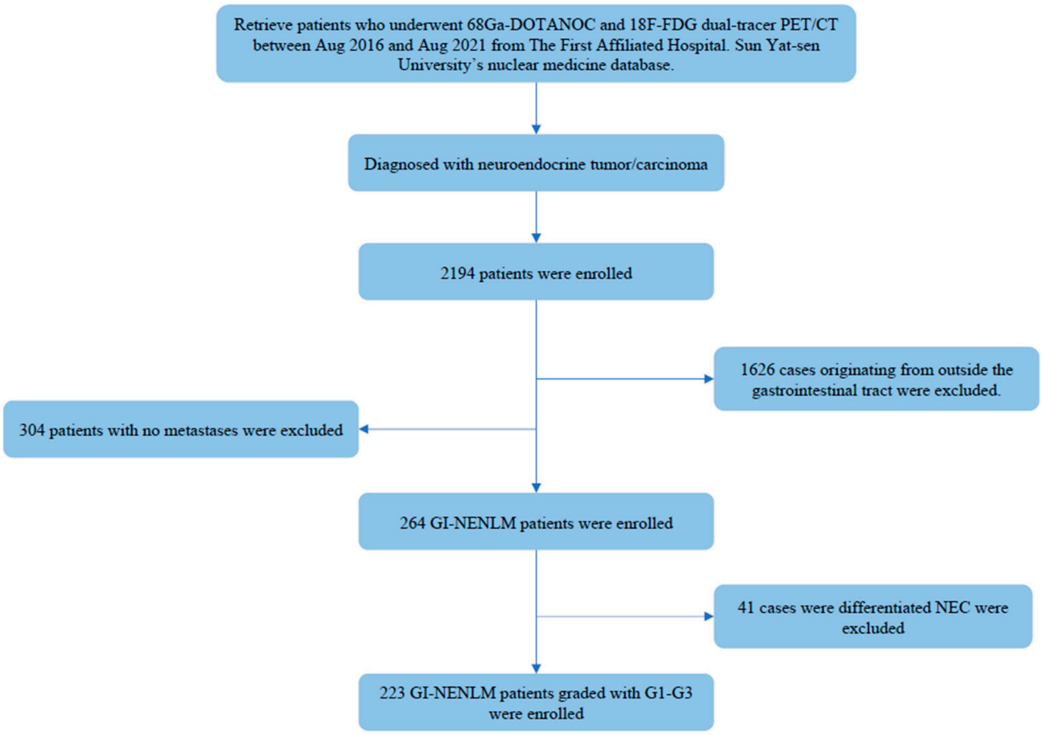

Figure S1. Patient Selection Flowchart.

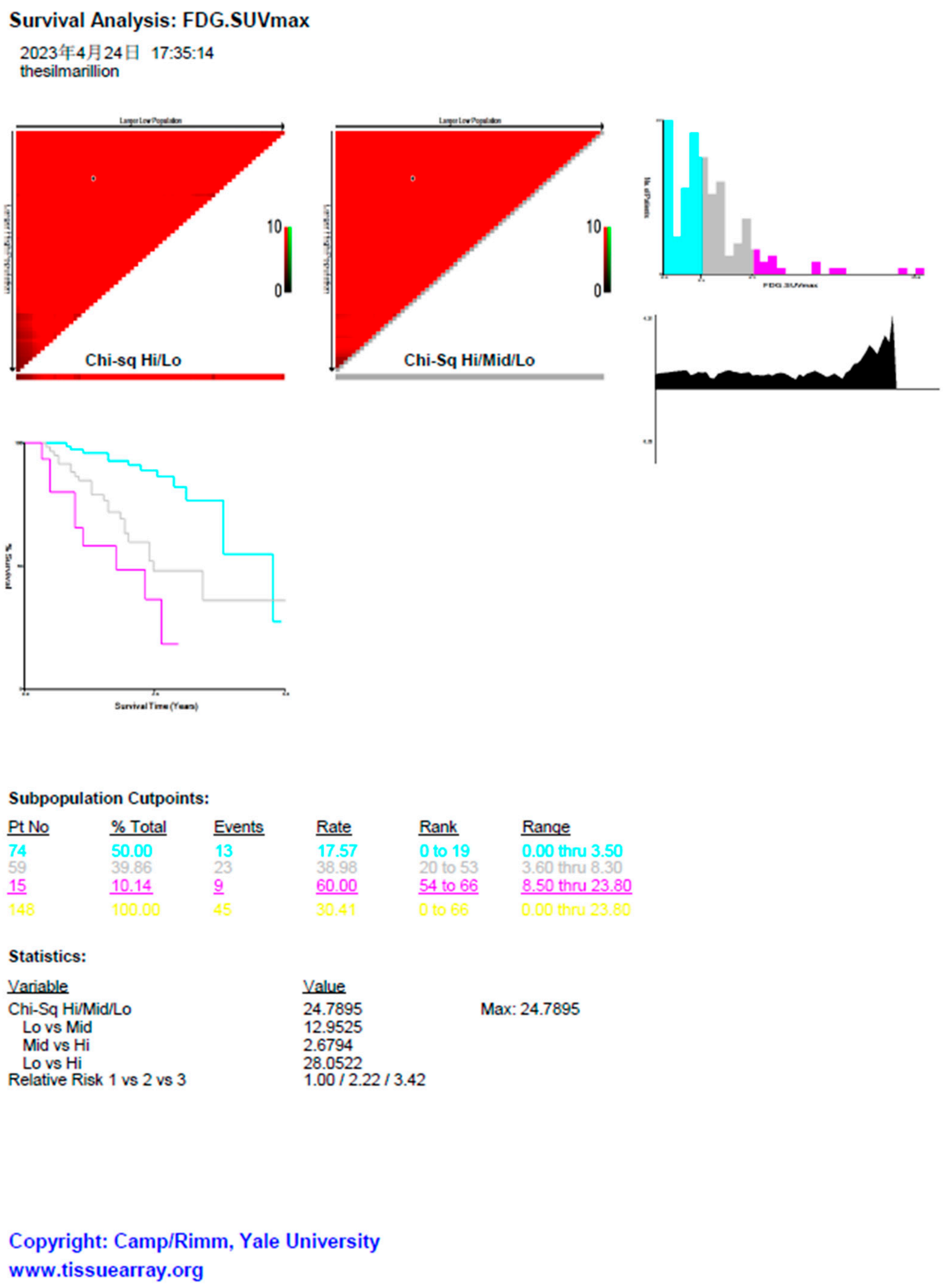

Pt No	% Total	Events	Rate	Rank	Range
74	50.00	13	17.57	0 to 19	0.00 thru 3.50
59	39.86	23	38.98	20 to 53	3.60 thru 8.30
15	10.14	9	60.00	54 to 66	8.50 thru 23.80
148	100.00	45	30.41	0 to 66	0.00 thru 23.80

Variable	Value	
Chi-Sq Hi/Mid/Lo	24.7895	Max: 24.7895
Lo vs Mid	12.9525	
Mid vs Hi	2.6794	
Lo vs Hi	28.0522	
Relative Risk 1 vs 2 vs 3	1.00 / 2.22 / 3.42	

Figure S2. The process of X-tile software for stratification of 18F FDG SUVmax values.

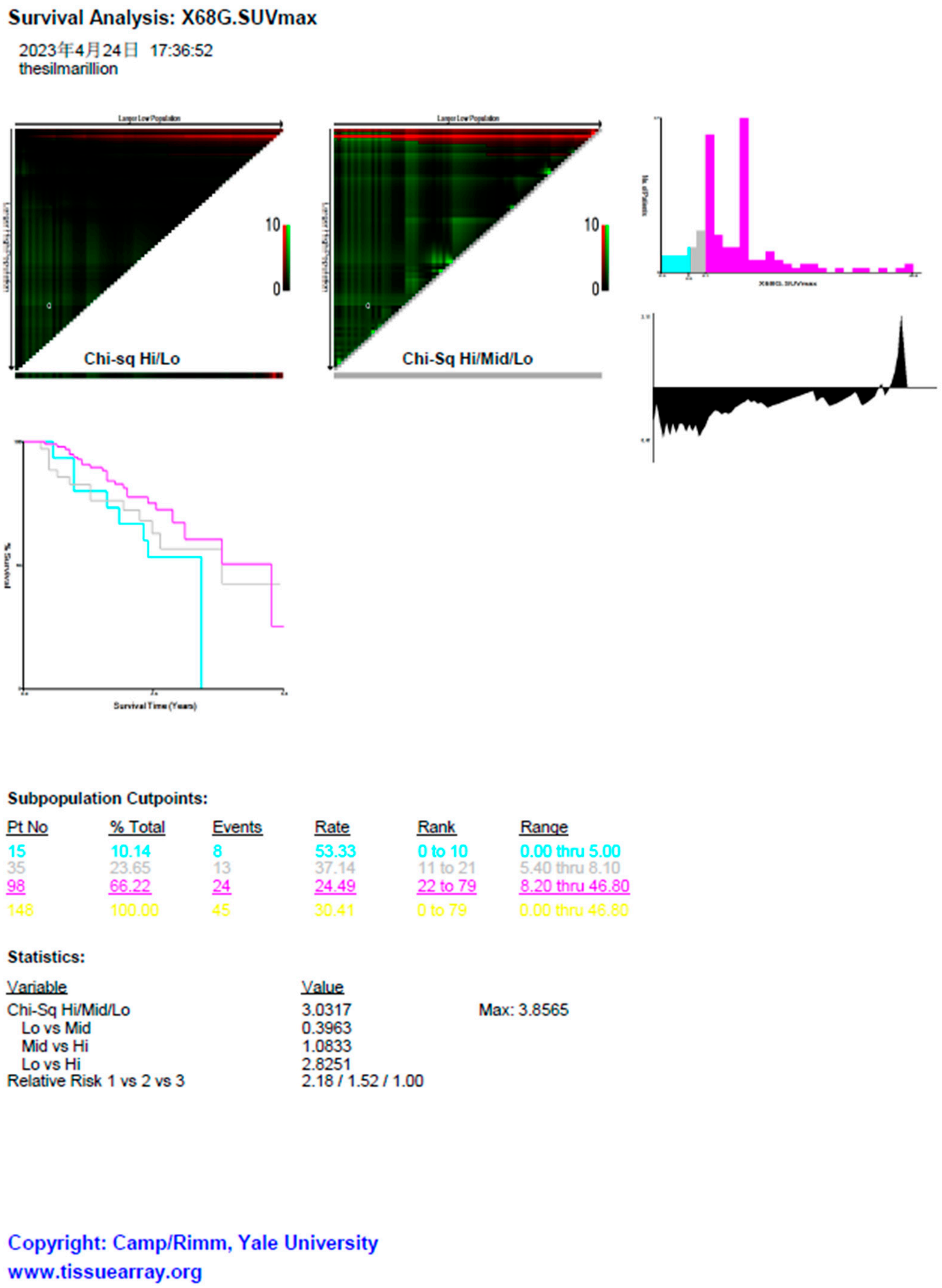

Figure S3. The process of X-tile software for stratification of FDG 68G DOTANOC SUVmax values.

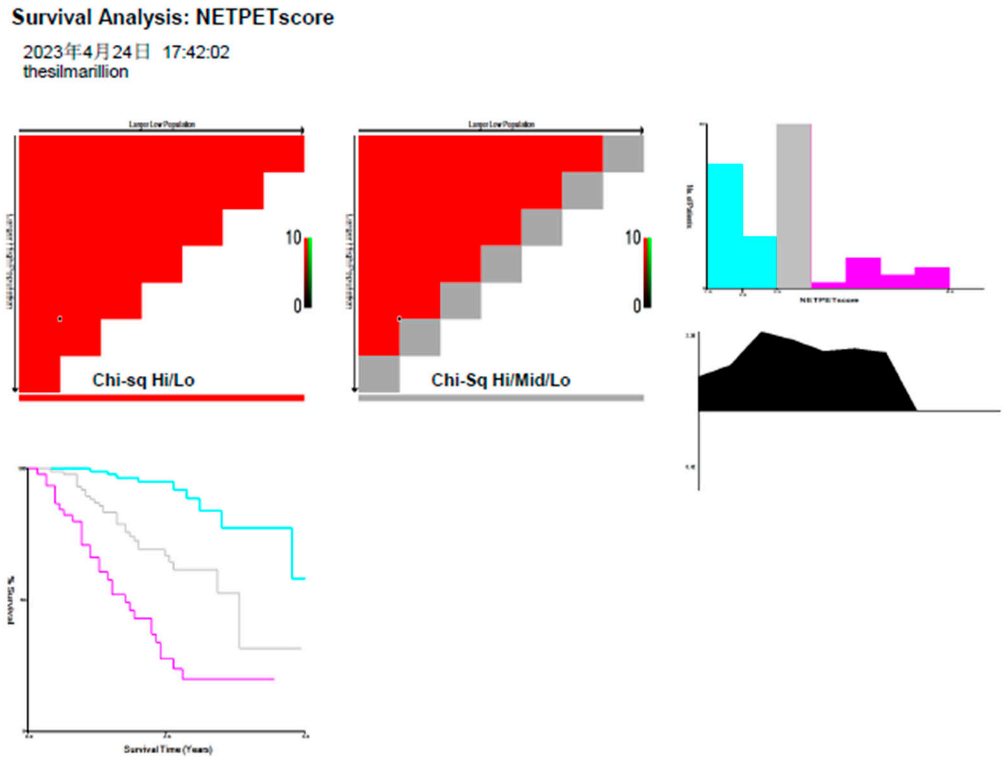

Subpopulation Cutpoints:

Pt No	% Total	Events	Rate	Rank	Range
92	41.26	9	9.78	0 to 1	1.00 thru 2.00
86	38.57	29	33.72	2 to 2	3.00 thru 3.00
45	20.18	30	66.67	3 to 7	4.00 thru 8.00
223	100.00	68	30.49	0 to 7	1.00 thru 8.00

Statistics:

Variable	Value	
Chi-Sq Hi/Mid/Lo	66.8310	Max: 66.8310
Lo vs Mid	18.8911	
Mid vs Hi	17.3412	
Lo vs Hi	68.5812	
Relative Risk 1 vs 2 vs 3	1.00 / 3.45 / 6.81	

Copyright: Camp/Rimm, Yale University
www.tissuearray.org

Figure S4. The process of X-tile software for stratification of NETPET score.

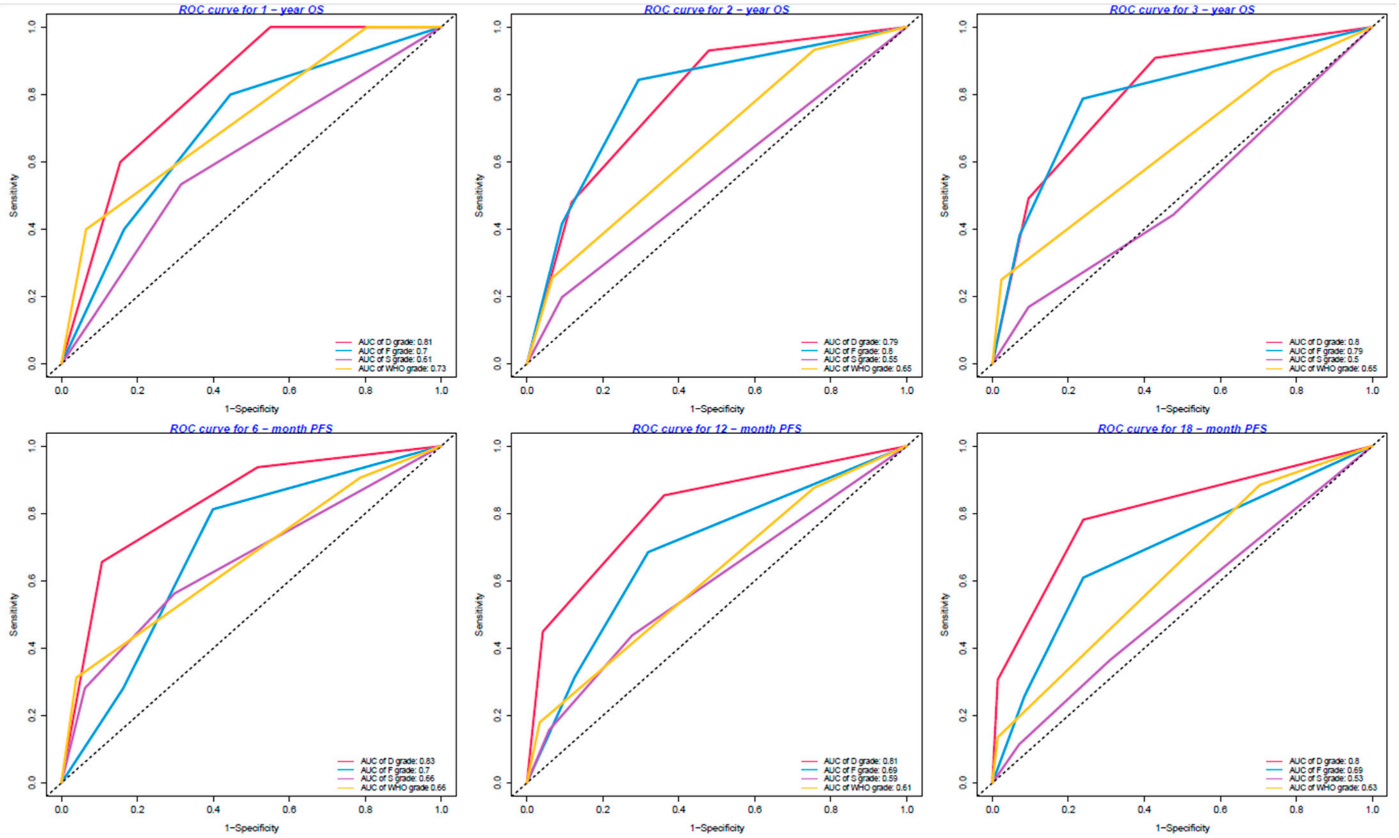

Figure S5. ROC curves for dual 18F-FDG and 68Ga DOTANOC PETCT visual grade (D grade) and examples of D grade, semiquantitative 18F-FDG PETCT grade (F grade), and semiquantitative 68Ga-DOTANOC PETCT grade (S grade) predicting 1-, 2-, 3-year OS and 6-, 12-, 18-month PFS in the overall cohort.

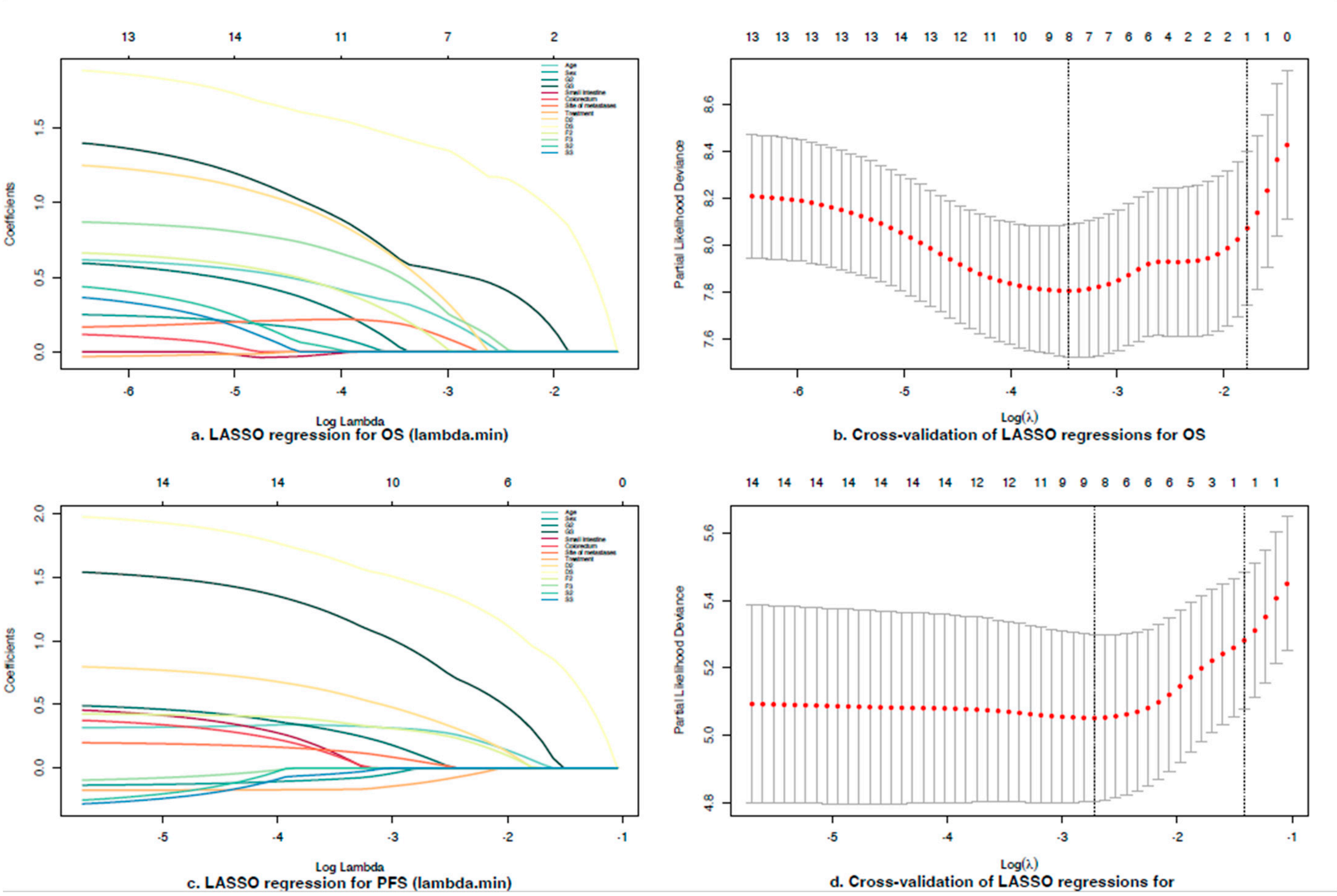

Figure S6. LASSO regression for OS and PFS. **a** LASSO regression for OS. **b** the cross-validation for LASSO regression for OS (lambada min). **c** LASSO regression for PFS. **d** the cross-validation for LASSO regression for PFS (lambada min).

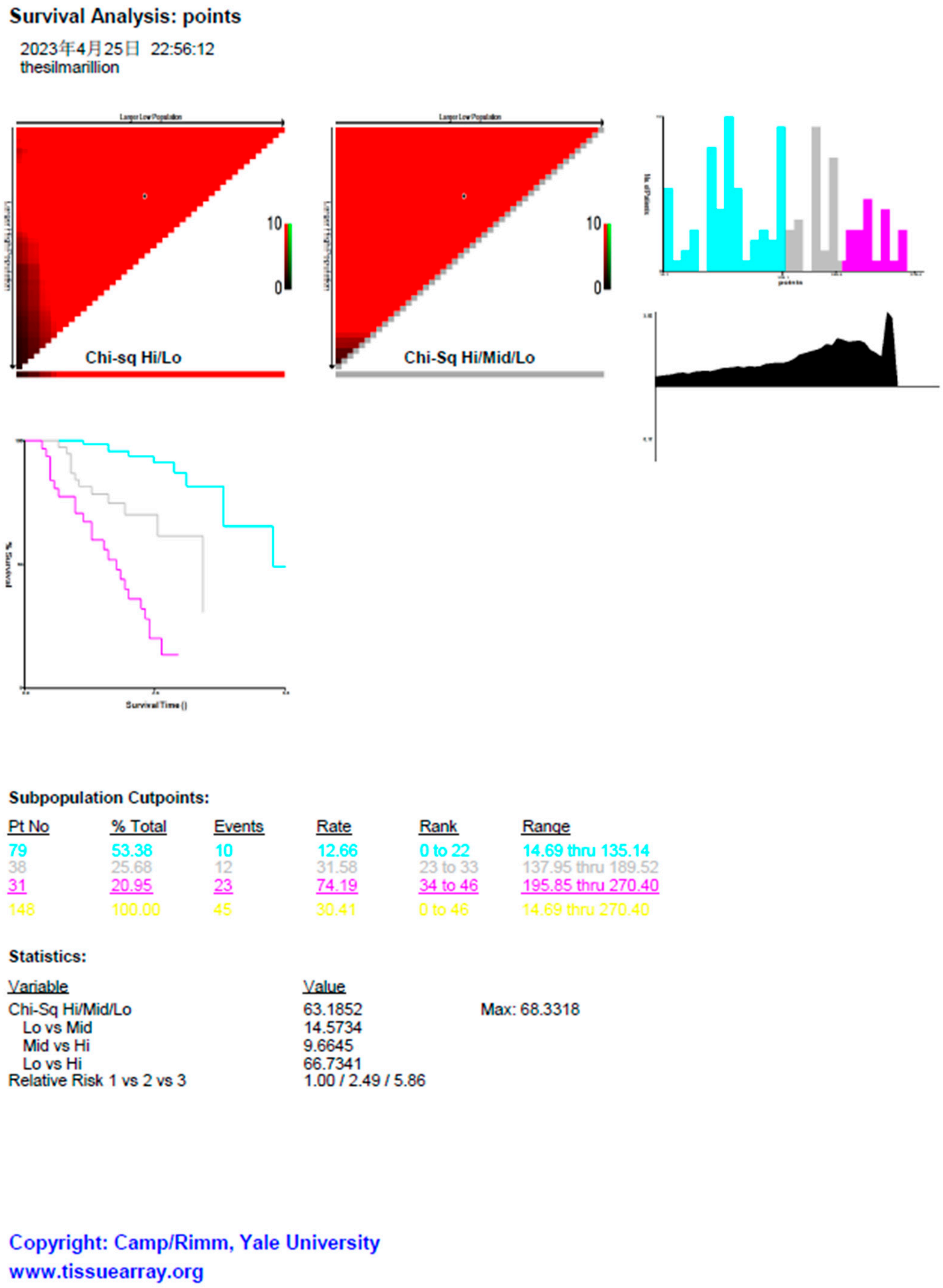

Subpopulation Cutpoints:

Pt No	% Total	Events	Rate	Rank	Range
79	53.38	10	12.66	0 to 22	14.69 thru 135.14
38	25.68	12	31.58	23 to 33	137.95 thru 189.52
31	20.95	23	74.19	34 to 46	195.85 thru 270.40
148	100.00	45	30.41	0 to 46	14.69 thru 270.40

Statistics:

Variable	Value	
Chi-Sq Hi/Mid/Lo	63.1852	Max: 68.3318
Lo vs Mid	14.5734	
Mid vs Hi	9.6645	
Lo vs Hi	66.7341	
Relative Risk 1 vs 2 vs 3	1.00 / 2.49 / 5.86	

Figure S7. The process of X-tile software for stratification of nomogram output scores for OS.

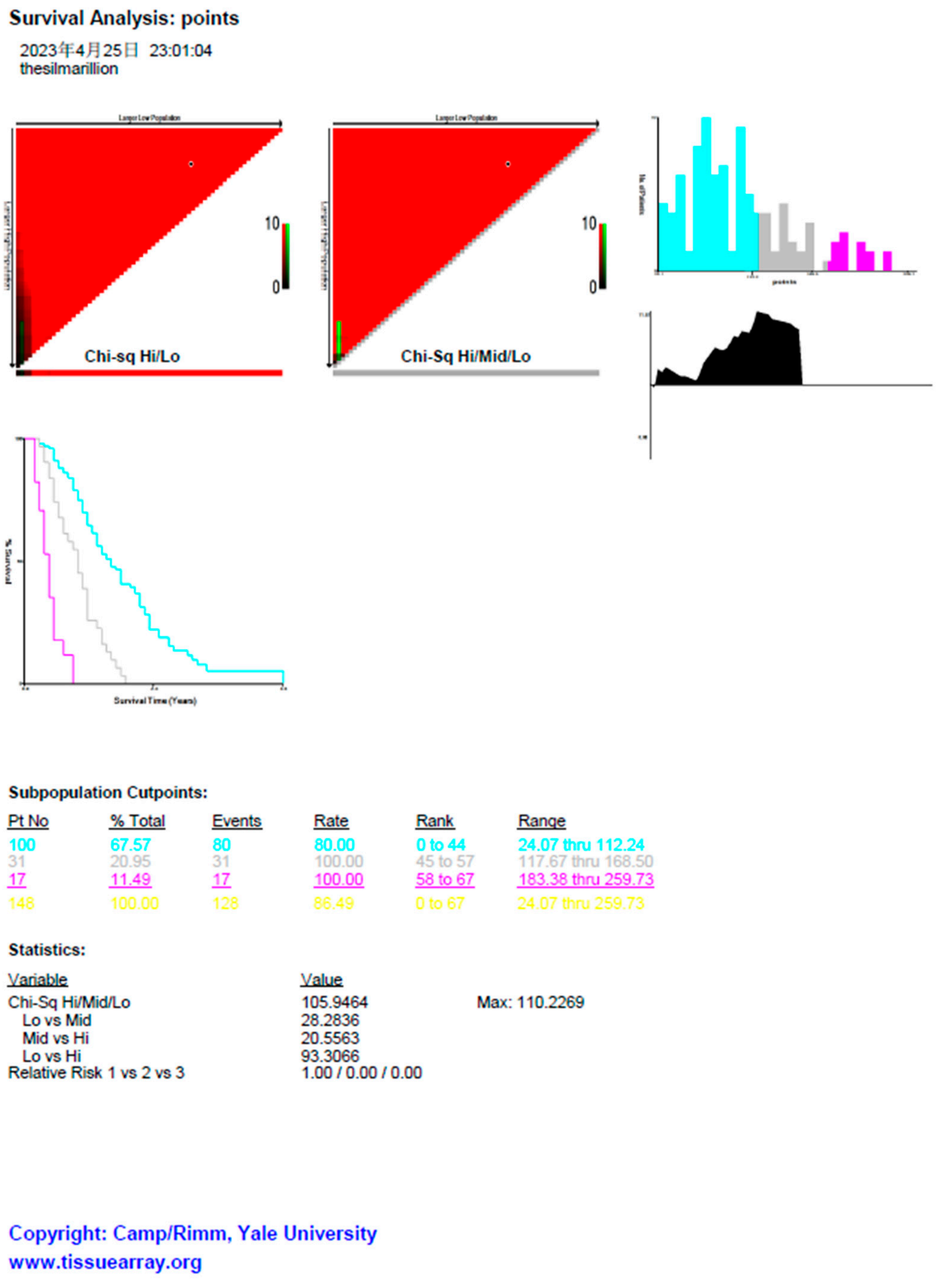

Figure S8. The process of X-tile software for stratification of nomogram output scores for PFS.

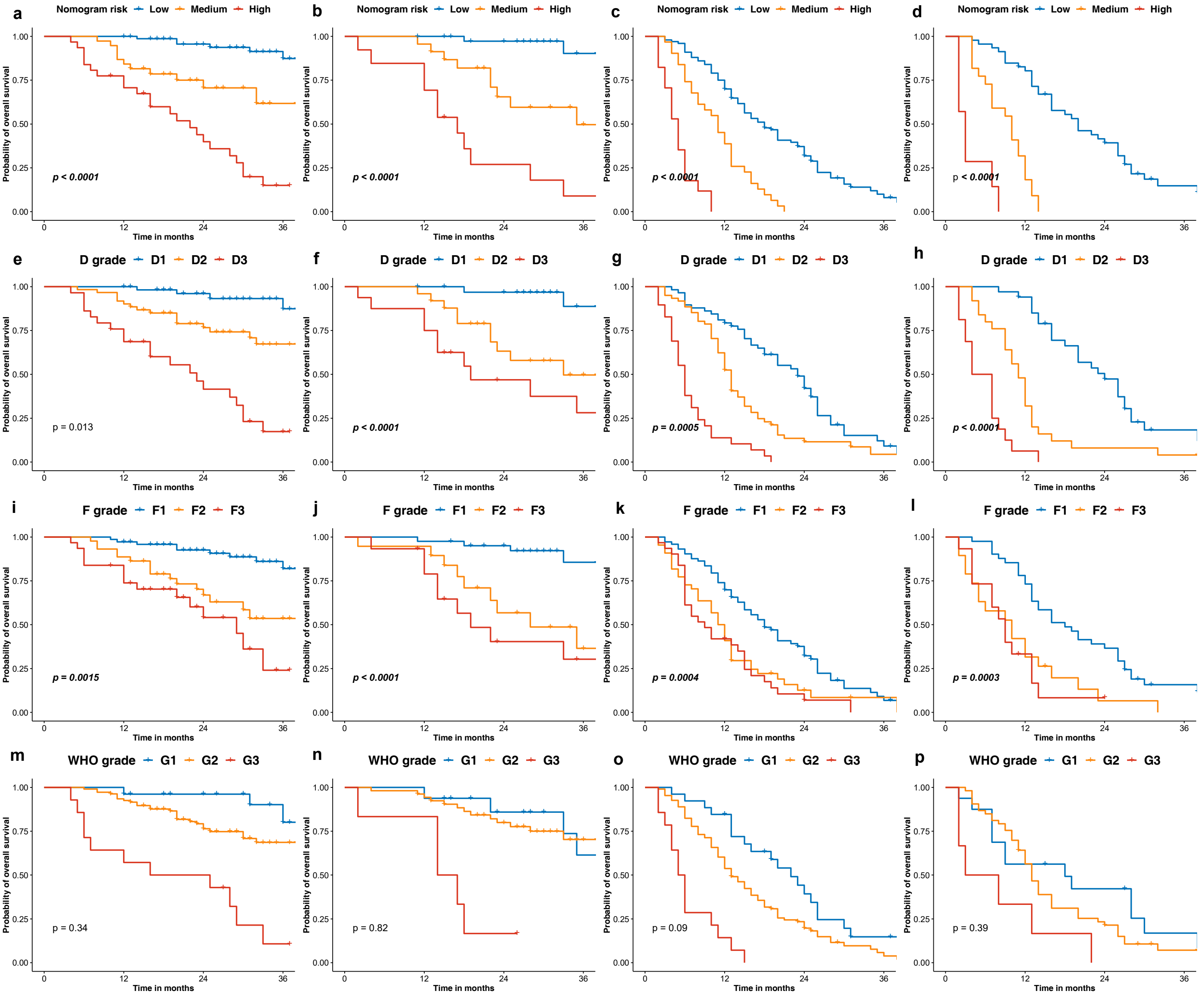

Figure S9. (a-b) The Kaplan Meier curves for risk Stratification Scale of the nomogram for OS in the training and internal validation cohort. **(c-d)** the Kaplan Meier curves for risk Stratification Scale of the nomogram for PFS in the training and internal validation cohort. **(e-f)** the Kaplan Meier curves for dual-scan visual grading for OS in the training and internal validation cohort. **(g-h)** the Kaplan Meier curves for dual-scan visual grading for PFS in the training and internal validation cohort. **(i-j)** the Kaplan Meier curves for semiquantitative 18F-FDG PETCT grading system for OS in the training and internal validation cohort. **(k-l)** the Kaplan Meier curves for semiquantitative 18F-FDG PETCT grading system for PFS in the training and internal validation cohort. **(m-n)** the Kaplan Meier curves for the WHO grading system for OS in the training and internal validation cohort. **(o-p)** the Kaplan Meier curves for the WHO grading system for PFS in the training and internal validation cohort.

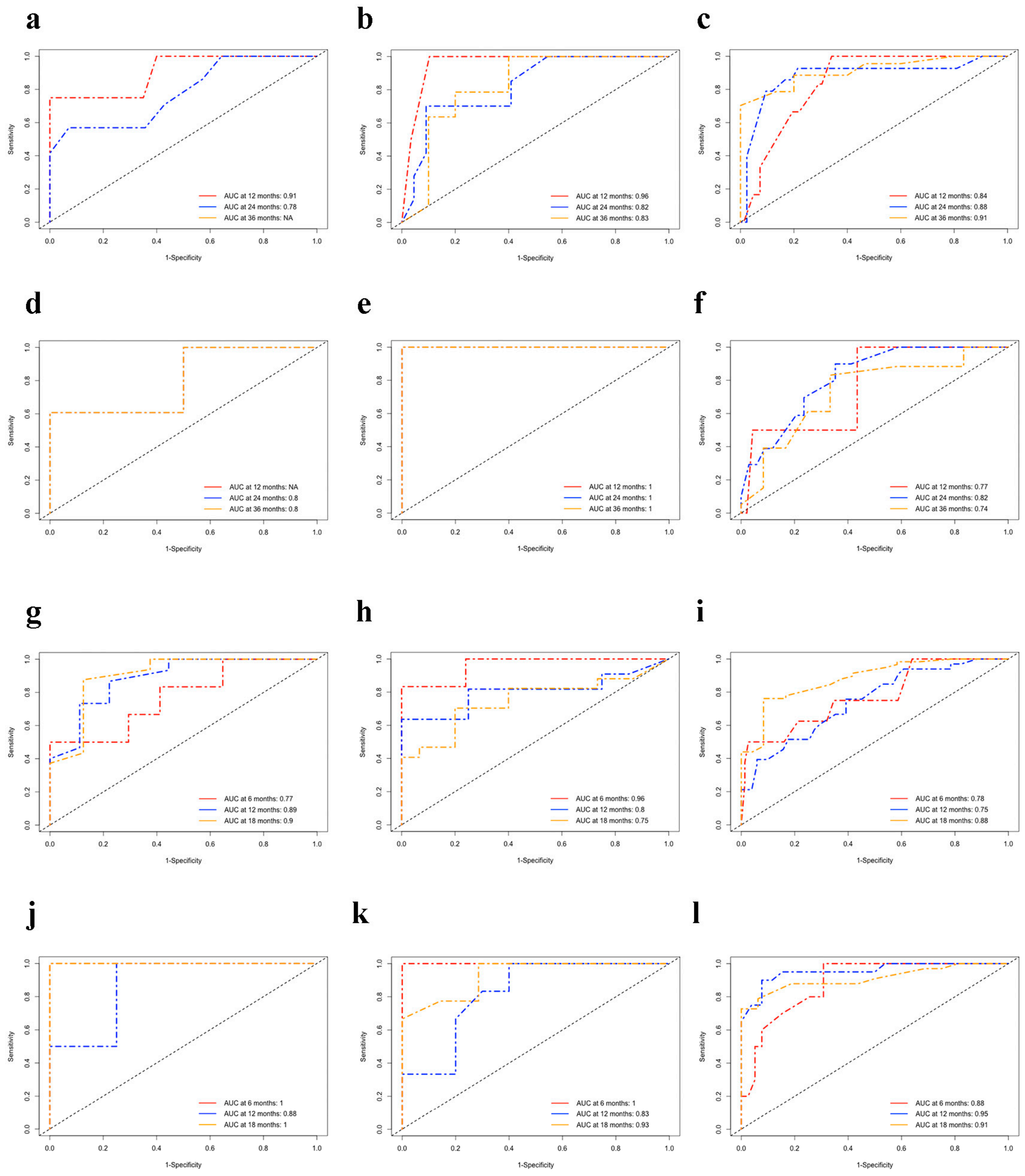

Figure S10. (a-l). Time ROC curve for OS. a The subgroup of stomach in Modelling cohort; b The subgroup of small intestine in Modelling cohort; c The subgroup of colorectum in Modelling cohort; d The subgroup of stomach in internal test cohort; e The subgroup of small intestine in internal test cohort; f The subgroup of colorectum in internal test cohort. Time ROC curve for PFS; g the subgroup of stomach in Modelling cohort; h The subgroup of small intestine in Modelling cohort; i The subgroup of colorectum in Modelling cohort; j The subgroup of stomach in internal test cohort; k The subgroup of small intestine in internal test cohort; l The subgroup of colorectum in internal test cohort.

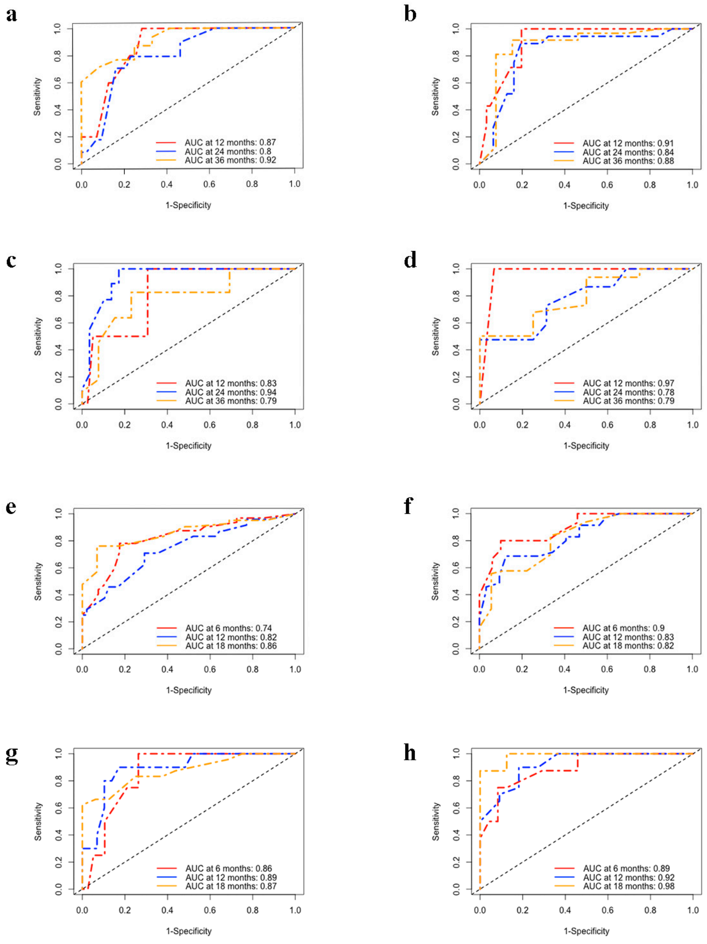

Figure S11. Time ROC curve for OS. a The subgroup of post-surgery in Modeling cohort; b The subgroup of no-surgery in Modeling cohort; c The subgroup of post-surgery in internal test cohort; d The subgroup of no-surgery in internal test cohort. Time ROC curve for PFS. e The subgroup of post-surgery in Modeling cohort; f The subgroup of no-surgery in Modeling cohort; g The subgroup of post-surgery in internal test cohort; h The subgroup of no-surgery in internal test cohort.
